# Heterologous functional expression of ascidian Nav1 channels and close relationship with the evolutionary ancestor of vertebrate Nav channels

**DOI:** 10.1016/j.jbc.2021.100783

**Published:** 2021-05-14

**Authors:** Takafumi Kawai, Masaki Hashimoto, Natsuki Eguchi, Junko M. Nishino, Yuka Jinno, Risa Mori-Kreiner, Måns Aspåker, Daijiro Chiba, Yukio Ohtsuka, Akira Kawanabe, Atsuo S. Nishino, Yasushi Okamura

**Affiliations:** 1 Integrative Physiology, Department of Physiology, Graduate School of Medicine, Osaka University, Suita, Japan; 2 Graduate School of Frontier Bioscience, Osaka University, Suita, Japan; 3 UC Davis, Davis, California, USA; 4 Department of Biology, Faculty of Agriculture and Life Science, Hirosaki University, Hirosaki, Japan; 5 Department of Bioresources Science, United Graduate School of Agricultural Sciences, Iwate University, Hirosaki, Japan; 6 Uppsala University, Uppsala, Sweden; 7 Biomedical Research Institute, National Institute of Advanced Industrial Science and Technology (AIST), Tsukuba, Ibaraki, Japan

**Keywords:** evolution, ion channel, sodium channel, ankyrin, axon initial segment, voltage clamp, cut-open oocyte, inactivation, ascidian, chordate, ABM, ankyrin-binding motif, AIS, axon initial segment, anti-MAP2, anti–microtubule-associated protein 2, AP, alkaline phosphatase, cDNA, complementary DNA, CiNav1a, ascidian *Ciona* Nav1, cRNA, complementary RNA, DDBJ, DNA Data Bank of Japan, DIG, digoxigenin, hβ1, human β1 subunit, HEK293T, human embryonic kidney 293T, Hepes, 4-(2-hydroxyethyl)-1-piperazineethanesulfonic acid, hNav1.5, human Nav1.5, I–V, current–voltage, Nav, voltage-gated sodium channel, PBST, PBS containing 0.1% Tween-20, PDB, Protein Data Bank, rNav1.4, rat Nav1.4, SSC, saline sodium citrate, TEVC, two-electrode voltage clamp, TTX, tetrodotoxin

## Abstract

Voltage-gated sodium channels (Nav1s) are responsible for the initiation and propagation of action potentials in neurons, muscle, and endocrine cells. Many clinically used drugs such as local anesthetics and antiarrhythmics inhibit Nav1s, and a variety of inherited human disorders are caused by mutations in Nav1 genes. Nav1s consist of the main α subunit and several auxiliary β subunits. Detailed information on the structure–function relationships of Nav1 subunits has been obtained through heterologous expression experiments and analyses of protein structures. The basic properties of Nav1s, including their gating and ion permeation, were classically described in the squid giant axon and other invertebrates. However, heterologous functional expression of Nav1s from marine invertebrates has been unsuccessful. Ascidians belong to the Urochordata, a sister group of vertebrates, and the larval central nervous system of ascidians shows a similar plan to that of vertebrates. Here, we report the biophysical properties of ascidian *Ciona* Nav1 (CiNav1a) heterologously expressed in *Xenopus* oocytes. CiNav1a exhibited tetrodotoxin-insensitive sodium currents with rapid gating kinetics of activation and inactivation. Furthermore, consistent with the fact that the *Ciona* genome lacks orthologous genes to vertebrate β subunits, the human β1 subunit did not influence the gating properties when coexpressed with CiNav1a. Interestingly, CiNav1a contains an ankyrin-binding motif in the II–III linker, which can be targeted to the axon initial segment of mammalian cortical neurons. Our findings provide a platform to gain insight into the evolutionary and biophysical properties of Nav1s, which are important for the development of targeted therapeutics.

Voltage-gated sodium channels (Nav1s) play a crucial role in not only membrane excitability in the nervous system but also muscle and endocrine cells. Nav1s are composed of a main (α) subunit and associated auxiliary (β1–4) subunits. The α subunit is comprised of four homologous domains, with each containing a voltage sensor, and a pore that is highly selective for sodium ions—a selectivity for sodium over 1000 times higher than for potassium and calcium ions. To date, there are ten Nav1 α subunit genes identified in mammals with their expressions regulated in tissue-specific and cell type–specific manner (1).

Nav1s were first characterized in the squid giant axon by Hodgkin and Huxley (2), who established the fundamental concepts in membrane excitability: “ion-selective pore” and “voltage sensor.” Since then, the basic properties of Nav1s, including their gating kinetics and ion permeation, have also been characterized in other invertebrates, such as snail neurons as well as the eggs of starfish and ascidians, which were advantageous to use because of their large cell size in two-electrode voltage clamp (TEVC) experiments, during a time before the age of patch clamping (3). Our understanding of Nav1 physiology continues to expand today with detailed information obtained through electrophysiological studies in heterologous expression combined with site-directed mutagenesis (1, 4, 5), analyses of human genetic disorders (6), and structural analyses using X-ray crystallography (4) and cryo-EM (7, 8, 9). Among cloned invertebrate Nav1s (10, 11, 12, 13, 14), the fly Nav, Para, has been successful in heterologous expression systems. Heterologous functional expression of sodium channels from marine invertebrates, including that of the squid giant axon, has been unsuccessful so far. Perhaps this is due to the seawater environment, which consists of electrolytes and osmolarity, that differ from the serum of vertebrates including *Xenopus*.

*Ciona*, an ascidian (or sea squirt), is a classic model system in developmental biology with attractive features that not only includes its mosaic embryogenesis and determinant factors in embryonic cell differentiation that precociously organizes in fertilized eggs but also for being universally available along coasts (15). *Ciona* is an excellent model system to study gene regulatory networks in cell differentiation (16) and also to gain insight into the vertebrate origins of neurogenic placodes (17), neural stem cell (18), neuronal subtypes (17, 19), and central nervous system connectome (20). Ascidians, which are members of the subphylum Tunicata, are located in a unique phylogenetic position, where they belong to the lineage of chordates and are considered more closely related to the ancestral vertebrate than the other group of chordates, the cephalochordates (amphioxus) (21). Of note, extensive work from the global analysis of the *Ciona* genome and expressed sequence tag provides us with the nucleic acid information on *Ciona robusta* (*Ciona intestinalis* type A) (22). A comprehensive analysis of the *Ciona* genome (23, 24) led to the identification of voltage-sensing phosphatase (25) and voltage-gated proton channel (Hv1/VSOP) (26).

Previous analysis of the *Ciona* genome identified three Nav1 α-like subunit genes, with one harboring structural characteristics conserved in vertebrate Nav1s, including ion-selectivity signature (D/E/K/A), inactivation latch sequence of the III–IV linker, and ankyrin-binding motif (ABM) of the cytoplasmic II–III linker (24, 27). This suggests that gene diversification for multiple Nav1 channel genes occurred after the branching point of ascidians and vertebrates (27). A series of classic electrophysiological studies on a different ascidian, *Halocynthia*, identified Nav1 complementary DNA (cDNA) (originally called TuNa1) expressed exclusively in neurons, which was considered as the prototype of ancestral vertebrate neuronal Nav1s (12). Gene expression of TuNa1 in neuronal precursors requires early cell contact by neighboring blastomere of inductive potential (12) that can be mimicked by basic fibroblast growth factor signal (28). However, heterologous functional expression of TuNa1 has been unsuccessful.

Here, we describe biophysical properties of *Ciona* Nav1 (renamed in this article as CiNav1a; previously named Ci-Nav1 (24)), the ortholog of TuNa1, heterologously expressed in *Xenopus* oocyte. CiNav1a showed tetrodotoxin (TTX)-insensitive sodium currents with very rapid gating of activation and inactivation. Coexpression of human β1 (hβ1) subunit did not affect the gating properties of CiNav1a, consistent with the fact that *Ciona* genome lacks orthologous gene to vertebrate β subunit genes (24). CiNav1a contains an ABM in the II–III linker, which can be targeted to the axon initial segment (AIS) of mammalian cortical neurons. These findings provide a new platform to gain novel insights into the evolutionary and biophysical properties of Nav1s.

## Results

### Primary structure of the ascidian Nav1, CiNav1a

**Figure 1:**
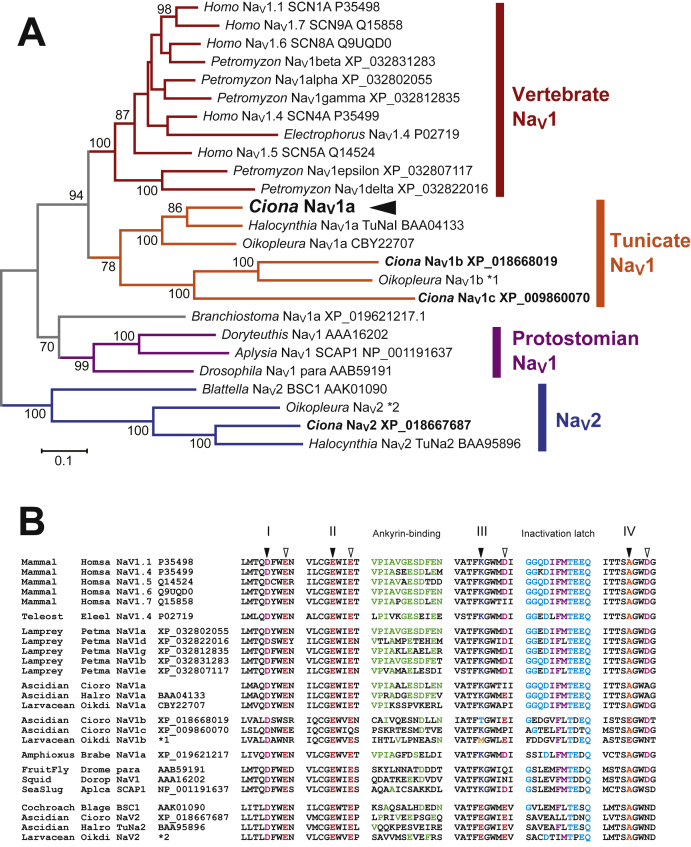
**Molecular phylogenetic characteristics of ascidian Nav1 homologs.***A*, a molecular phylogenetic tree of Nav1 α subunits. Sequences with homology were collected from public databases to prepare a gap-free alignment of 1148 amino acids. The “maximum likelihood” tree with the highest log likelihood is depicted. The results of 100 replicates of bootstrap analysis were also depicted, only when the values were larger than 70. National Center for Biotechnology Information accession numbers of the sequences and genus names from which the sequences were derived are shown. Nav2 channel sequences were used as outgroup. *Ciona* has three Nav1-like sequences that are closely related to vertebrate Nav1s. ∗1 and ∗2 indicate the sequences that have been identified as Nav1-like gene models in *Oikopleura dioica* (appendicularian tunicate) genome database (https://www.aniseed.cnrs.fr/). Gene model IDs of ∗1 and ∗2 are OD_K25COV10_DN16743_c0_g1_i1 and OD_K25COV10_DN18236_c0_g1_i7, respectively. *Ciona* Nav1a is highlighted with *arrowhead*. *B*, consensus amino acid sequences in critical regions, including pore turrets in S5–S6 loops from domain I–IV that determine ion selectivity (*black arrowheads*) and another associated lining of acidic amino acids (*white arrowheads*), ankyrin-binding motif region in the II–III linker, and inactivation latch in the III–IV linker. The amino acids at the pore turrets are highlighted with color (Asp [D], *magenta*; Glu [E], *red*; Lys [K], *blue*; Ala [A], *orange*; Thr [T], *light blue*; and Met [M], *yellow*). The core triplets of the inactivation latch (I–F–M) are marked in *purple*. Amino acids identical to the mammalian Nav1.1 ankyrin-binding motif are indicated by *green letters*. *Cyan residues* indicate the amino acids identical to those around the core triplet in the mammalian Nav1.1 inactivation latch. Nav, voltage-gated sodium channel.

Previous homology search from the *Ciona* genome identified four putative sodium channel genes (24). Subsequent comprehensive search for sodium channel genes in the updated *Ciona* genome (29) reconfirmed the four genes. Molecular phylogenetic analysis using the predicted amino acid sequences of the identified genes revealed that the three paralogs, CiNav1a, CiNav1b, CiNav1c, are at a relatively close position to the clade of vertebrate Nav1 channels (Fig. 1*A*). The other one, CiNav2, is closely related to the clade of Nav2 including BSC1 of cockroach *Blattella* and TuNa2, the previously identified Nav channel–like gene from another ascidian *Halocynthia* (Fig. 1*A*) (27, 30). CiNav1a (former name Ci-Nav1 in (24)) not only contains sequences highly homologous to vertebrate Nav1s but also showed the highest homology to the previously identified TuNa1 from *Halocynthia*, a TTX-insensitive neuronal Nav channel (12). Using available cDNA and genomic information of *Ciona* (29, 31), we performed RT-PCR from *Ciona* tadpole larva to obtain the full-length CiNav1a cDNA. Because the full length is long, we obtained two (5’ and 3’) cDNA fragments for covering the full-length cDNA using two sets of PCR primers (see Experimental procedures section for details).

The deduced primary structure of CiNav1a was of 2323 amino acids. Critical regions for Nav1 functions are more evidently conserved in CiNav1a than in CiNav1b and CiNav1c (Fig. 1*B*). Those critical regions include the pore turrets for ion selectivity in the S5–S6 loop from each quadrant of the four repetitive domains (I–IV) with the signature D/E/K/A as found in other typical Nav1s, and also the S4 for voltage sensing, ABM in the II–III loop (32, 33), and III–IV linker for fast inactivation (Fig. 1*B*) (27, 34). Those critical regions were also conserved in TuNa1 (BAA04133) of *Halocynthia* and mostly in a putative ortholog (CBY22707) of *Oikopleura* (which belong to another tunicate group of ascidians). These characteristics suggest that CiNav1a represents an original chordate type of Nav1 that retains basic molecular functions inherited to all vertebrate Nav1 isoforms (Fig. 1).

### Ascidian Nav1, CiNav1a, showed TTX-insensitive sodium current with rapid gating in *Xenopus* oocyte

**Figure 2:**
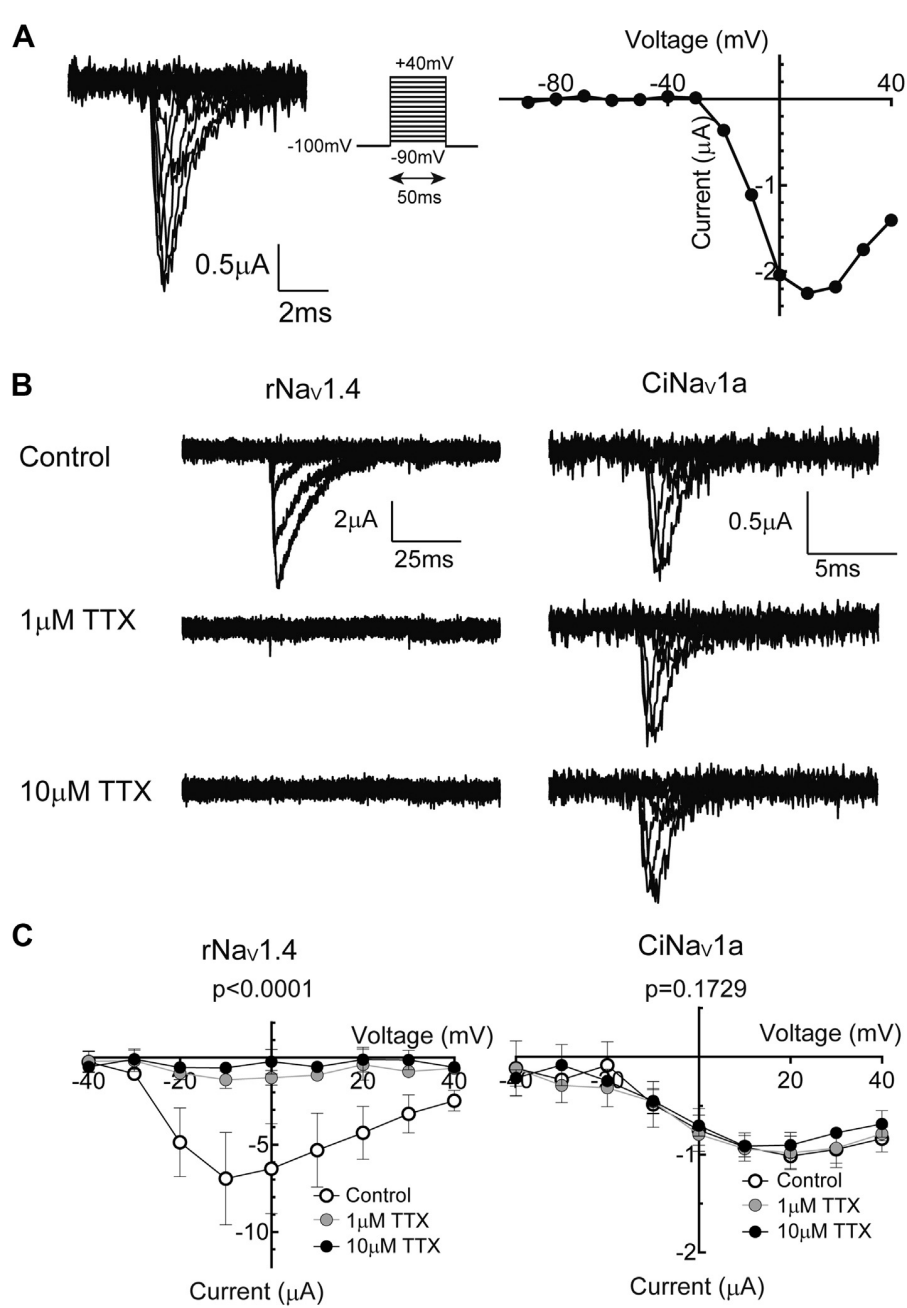
**TTX-insensitive sodium current from CiNav1a expressed in *Xenopus* oocyte.***A*, raw traces and *I*–*V* curve of CiNav1a by TEVC recording. Only cRNA encoding CiNav1a was microinjected into *Xenopus* oocyte. Holding potential was −100 mV, and depolarizing pulse in 50 ms was stepped to various levels (−90 to 40 mV). The current was isolated by leak subtraction using the P/4 protocol. *B*, representative TEVC recordings showing TTX insensitivity of the current of CiNav1a. CiNav1a is insensitive even to 10 μM TTX, whereas 1 μM TTX is enough to abolish rNav1.4 current. The pulse protocol is the same to *A*. *C*, *I*–*V* curves of CiNav1a and rNav1.4 current under no TTX, 1 μM TTX, or 10 μM TTX (N = 3 for each experiment). *p* < 0.0001 for rNav1.4. *p* = 0.1729 for CiNav1a as analyzed by two-way repeated-measures ANOVA. All graphs present mean ± SD. CiNav1a, ascidian *Ciona* Nav1; cRNA, complementary RNA; *I*–*V*, current–voltage; TEVC, two-electrode voltage clamp; TTX, tetrodotoxin.

Expressing full-length CiNav1a mRNA in *Xenopus* oocytes, we performed TEVC to record sodium currents. Cells that expressed CiNav1a exhibited typical voltage-gated sodium currents with rapid activation and inactivation (Fig. 2). CiNav1a is grouped in the same clade as the previously characterized ascidian sodium channel, TuNa1 from *Halocynthia roretzi* (Fig. 1*A*), which was reported to be TTX insensitive in native neural cells (12). Applying 10 μM TTX suppressed the peak inward current of CiNav1a expressed in *Xenopus* oocytes by less than 5%, whereas it completely inhibited currents of rat Nav1.4 (rNav1.4) (Fig. 2, *B* and *C*). This result indicates that CiNav1a is almost insensitive to TTX. CiNav1a cDNA transfected into human embryonic kidney 293T (HEK293T) cells failed to express functional Nav current (data not shown).

### Ascidian Nav1, CiNav1a, is insensitive to human Nav1 β1 subunit

In mammals, β subunits are known to increase cell surface expression and modify the gating properties of α subunits. To examine the effects of β subunit on CiNav1a, we performed cut-open oocyte recording and compared sodium currents between cells expressing only CiNav1a and cells coexpressing CiNav1a with hβ1. rNav1.4 (also called μ1) and human Nav1.5 (hNav1.5, also called hH1) were tested as positive controls known to be modulated by β1 subunits.

**Figure 3:**
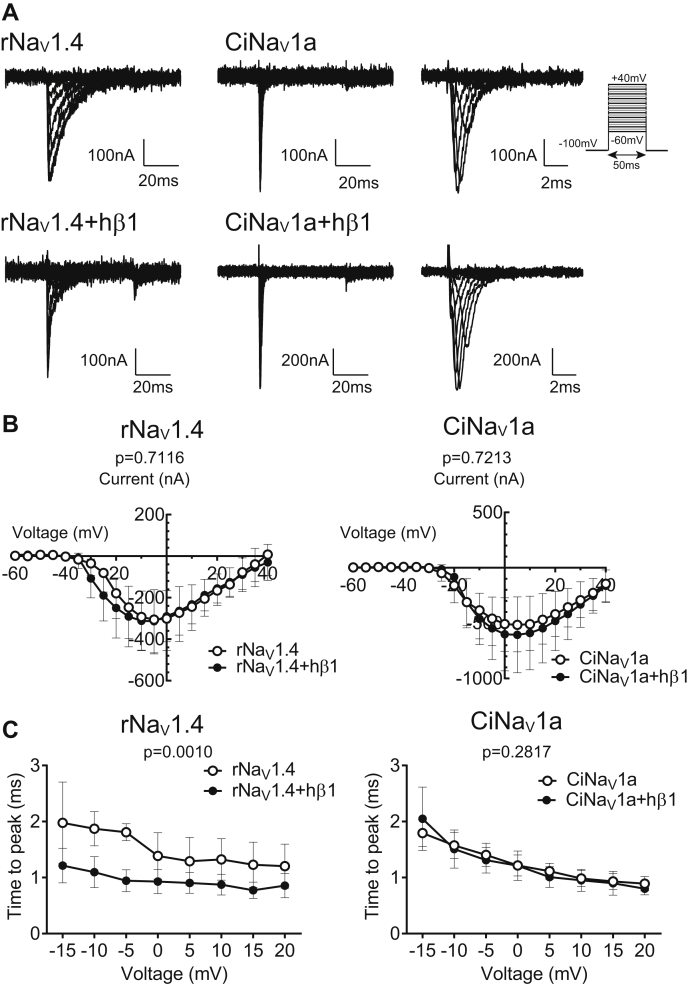
**Effect of human β1 subunit on activation properties of CiNav1a.***A*, representative traces of CiNav1a and rNav1.4 alone or with human β1 recorded by cut-open oocyte recording. Holding potential was −100 mV. Depolarizing step was elicited for 50 ms by 5 mV increment. Traces with 10 mV increment ranging from −60 to 40 mV are shown. The current was isolated by leak subtraction using the P/4 protocol. Noise was removed offline by Gaussian digital low-pass filter at cutoff frequency at 3 kHz. *B*, *I*–*V* curves of CiNav1a and rNav1.4 current with or without hβ1. N = 7, 6, 7, and 5 for rNav1.4 alone, rNav1.4 with hβ1, CiNav1a alone, and CiNav1a with hβ1, respectively. *p* = 0.7116 for rNav1.4. *p* = 0.7213 for CiNav1a as analyzed by two-way repeated-measures ANOVA. *C*, activation kinetics (time to peak) of CiNav1a and rNav1.4 plotted against membrane potential. All graphs present mean ± SD. N = 7, 6, 7, and 5 for rNav1.4 alone, rNav1.4 with hβ1, CiNav1a alone, and CiNav1a with hβ1, respectively. *p* = 0.0010 for rNav1.4. *p* = 0.2817 for CiNav1a as analyzed by two-way repeated-measures ANOVA. CiNav1a, ascidian *Ciona* Nav1; *I*–*V*, current–voltage; rNav1.4, rat Nav1.4.

**Figure 4:**
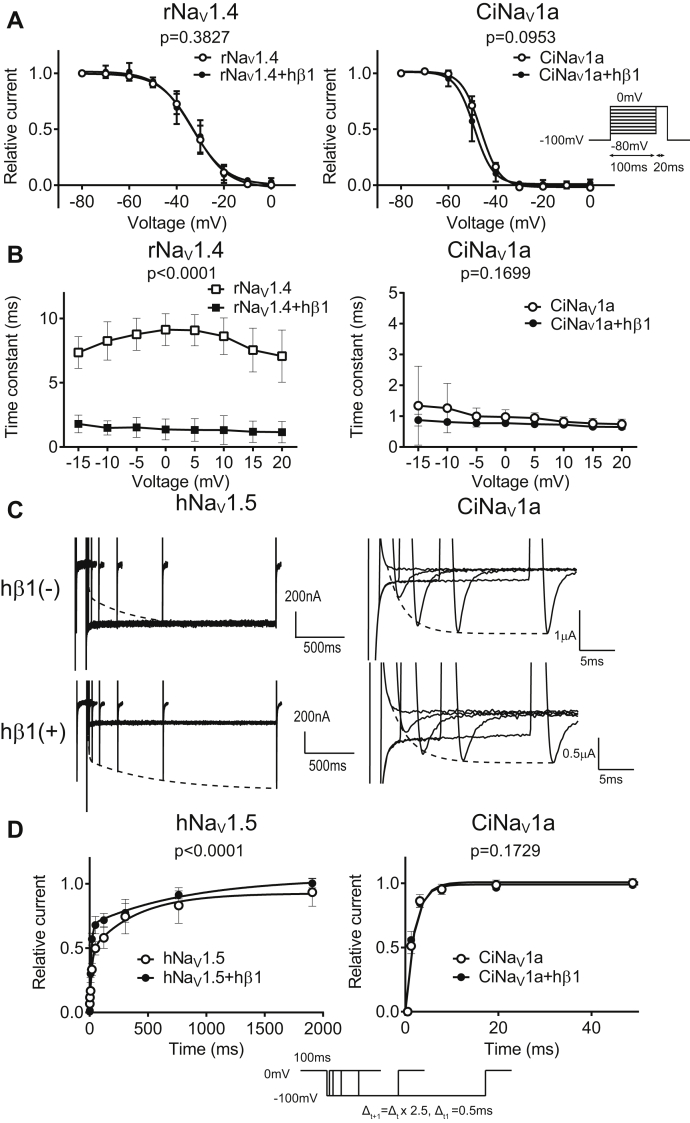
**Effect of hβ1 on inactivation properties of CiNav1a.***A*, effect of hβ1 on steady-state inactivation curve of CiNav1a as examined by cut-open oocyte recordings. rNav1.4 was also analyzed. Pulse protocol was as follows: interval, 200 ms; prepulse duration, 100 ms; test pulse duration, 20 ms; test pulse voltage, 0 mV; and holding potential, −100 mV. N = 5, 7, 7, and 4 for CiNav1a alone, CiNav1a + hβ1, rNav1.4 alone, and rNav1.4 + hβ1, respectively. *p* = 0.3827 for rNav1.4. *p* = 0.0953 for CiNav1a as analyzed by two-way repeated-measures ANOVA. *B*, inactivation kinetics (time constant of decay phase in fitting by single exponentials) of CiNav1a and rNav1.4 currents plotted against membrane potential as examined by cut-open oocyte recordings. Holding potential was −100 mV, and depolarizing pulse in 50 ms was stepped by 5 mV increment with 200 ms interval. The current was isolated by leak subtraction using the P/4 protocol. N = 7, 6, 7, and 5 for rNav1.4 alone, rNav1.4 with hβ1, CiNav1a alone, and CiNav1a with hβ1, respectively. Inactivation kinetics was significantly accelerated by coexpression with hβ1 in rNav1.4 (*p* < 0.0001), whereas there was no significant difference in time constant (*p* = 0.1699) in CiNav1a as analyzed by two-way repeated-measures ANOVA. *C*, representative traces of CiNav1a and hNav1.5 with or without hβ1 showing the kinetics of recovery from inactivation as examined by TEVC. Holding potential and interval potential was −100 mV. Preconditioning pulse was 0 mV for 100 ms for both types of channels. Test pulse was 0 mV for 50 ms for hNav1.5 and 100 ms for CiNav1a. *D*, recovery from inactivation with or without hβ1. Pulse protocol is shown. hNav1.5 current recovered from inactivation in two phases and fit by two exponential components, whereas CiNav1a current recovered in single phase and fit by single exponential component. Note that recovery from inactivation is accelerated by hβ1 in hNav1.5 (*p* < 0.0001, N = 5, 7 for hNav1.5 alone and with hβ1, respectively), whereas there was no significant difference (*p* = 0.1729) in CiNav1a (N = 10, 9 for CiNav1a alone and with hβ1, respectively) as analyzed by two-way repeated-measures ANOVA. Also note that some of the error bars are too small to be discerned in the graphs. CiNav1a, ascidian *Ciona* Nav1; hβ1, human β1 subunit; hNav1.5, human Nav1.5; rNav1.4, rat Nav1.4; TEVC, two-electrode voltage clamp.

To test the effects of hβ1, we first verified the functional expression of hβ1 by observing its known influences on the current decay of rNav1.4, which was remarkedly accelerated (Figs. 3*A* and 4*B*). In contrast, hβ1 did not alter the current decay kinetics of CiNav1a (Fig. 4*B*). The current–voltage (*I*–*V*) curve and activation speed (Fig. 3, *B* and *C* and Fig. S1) were not significantly different between cells only expressing CiNav1a and those coexpressing with hβ1. Steady-state inactivation of CiNav1a (Fig. 4*A*) as examined by recording in cut-open oocyte also showed no difference between the presence and absence of hβ1 (CiNav1a: V_half = −46.39 ± 0.2931 [mV]; *k* [slope factor] = 4.001 ± 0.2253 [mV]; N = 5; CiNav1a + hβ1: V_half = −49.03 ± 0.4525 [mV]; *k* = 3.958 ± 0.4790 [mV]; N = 7, as fitted with Boltzmann function [mean ± SD]). Consistent with previously reported data (35), fits of the recovery time course required two exponential components in rNav1.4. hβ1 accelerated the recovery from inactivation of hNav1.5 as examined at −100 mV (hNav1.5: tau1 = 20.64 ± 11.443 ms, tau2 = 868.666 ± 1057.546 ms, N = 5; hNav1.5 + hβ1: tau1 = 9.861 ± 1.455 ms, tau2 = 821.217 ± 442.856 ms, N = 7, in fitting with double exponential function [mean ± SD]), whereas no significant difference was observed in the recovery from inactivation in cells expressing CiNav1a with or without hβ1 (CiNav1a: tau = 2.151 ± 0.702 ms, N = 10; CiNav1a + hβ1: tau = 2.401 ± 0.687 ms, N = 9, in fitting with single exponential function [mean ± SD]) (tau1, tau2, tau are time constant of each exponential component) (Fig. 4, *C* and *D*). We also tried to coexpress CiNav1a with hβ1 in HEK293T cells but failed to observe functional currents.

### Ascidian Nav1, CiNav1a, showed insensitivities to insect Nav1 auxiliary subunit, TipE

**Figure 5:**
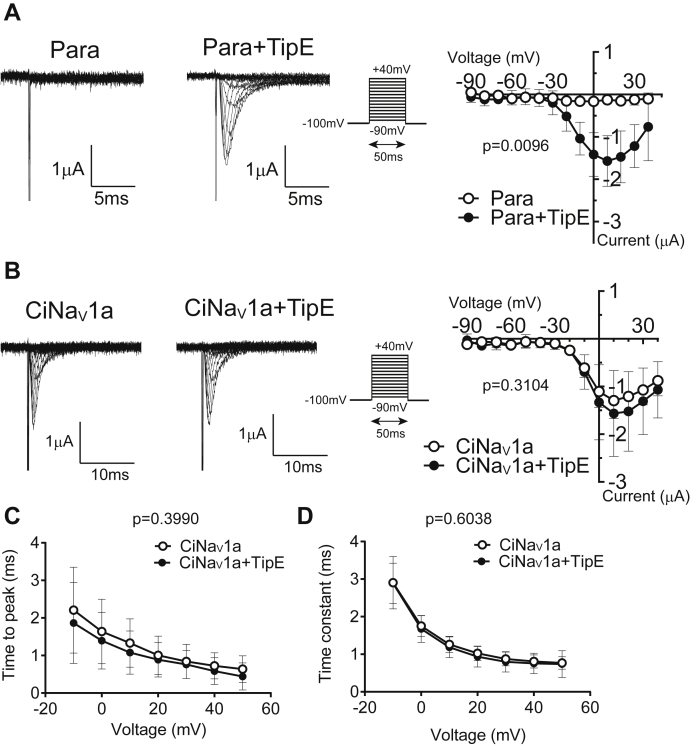
**Comparison of effect of TipE between CiNav1a and fly Para Nav channel studied by TEVC.***A*, TipE drastically increases the Para current (*p* = 0.0096). *Left*, representative current traces of Para with or without TipE. *Right*, *I*–*V* curve of Para current with or without TipE. N = 7 for each. Holding potential was −100 mV, and depolarizing step was applied for 50 ms by 10 mV increment ranging from −90 to 40 mV. *B*, TipE does not modulate CiNav1a current (*p* = 0.3104). *Left*, representative current traces of CiNav1a with or without TipE. *Right*, *I*–*V* curve of CiNav1a current with or without TipE. N = 12 and 13 for CiNav1a and CiNav1a with TipE, respectively. The pulse protocol is the same as in *A*. Raw traces elicited during steps from −90 to 60 mV are superimposed. In *A* and *C*, the current was isolated by leak subtraction using the P/4 protocol. The surge to the inward direction was due to leak subtraction. *C*, time to peak of CiNav1a current with or without TipE (*p* = 0.3990). *D*, decay time constant of CiNav1a current with or without TipE. Values in *C* and *D* are derived from single exponential fitting of the dataset shown in *A* and *B*. *p* = 0.6038. Two-way repeated-measures ANOVA was performed. All graphs present mean ± SD. CiNav1a, ascidian *Ciona* Nav1; *I*–*V*, current–voltage; Nav, voltage-gated sodium channel; TEVC, two-electrode voltage clamp.

Although ascidians are more closely related to vertebrates than to insects, we tested for possible effects of insect Nav1 auxiliary subunit, TipE (36), on the molecular functions of CiNav1a. TipE drastically enhanced the current expression of *Drosophila* Nav1 Para (Fig. 5*A*), whereas it did not change the current amplitude of CiNav1a (Fig. 5*B*). In addition, coexpression with TipE did not lead to any significant change of the *I*–*V* curve, activation speed, and decay kinetics of CiNav1a (Fig. 5, *B*–*D*).

### The ascidian Nav1 is exclusively expressed in neurons

**Figure 6:**
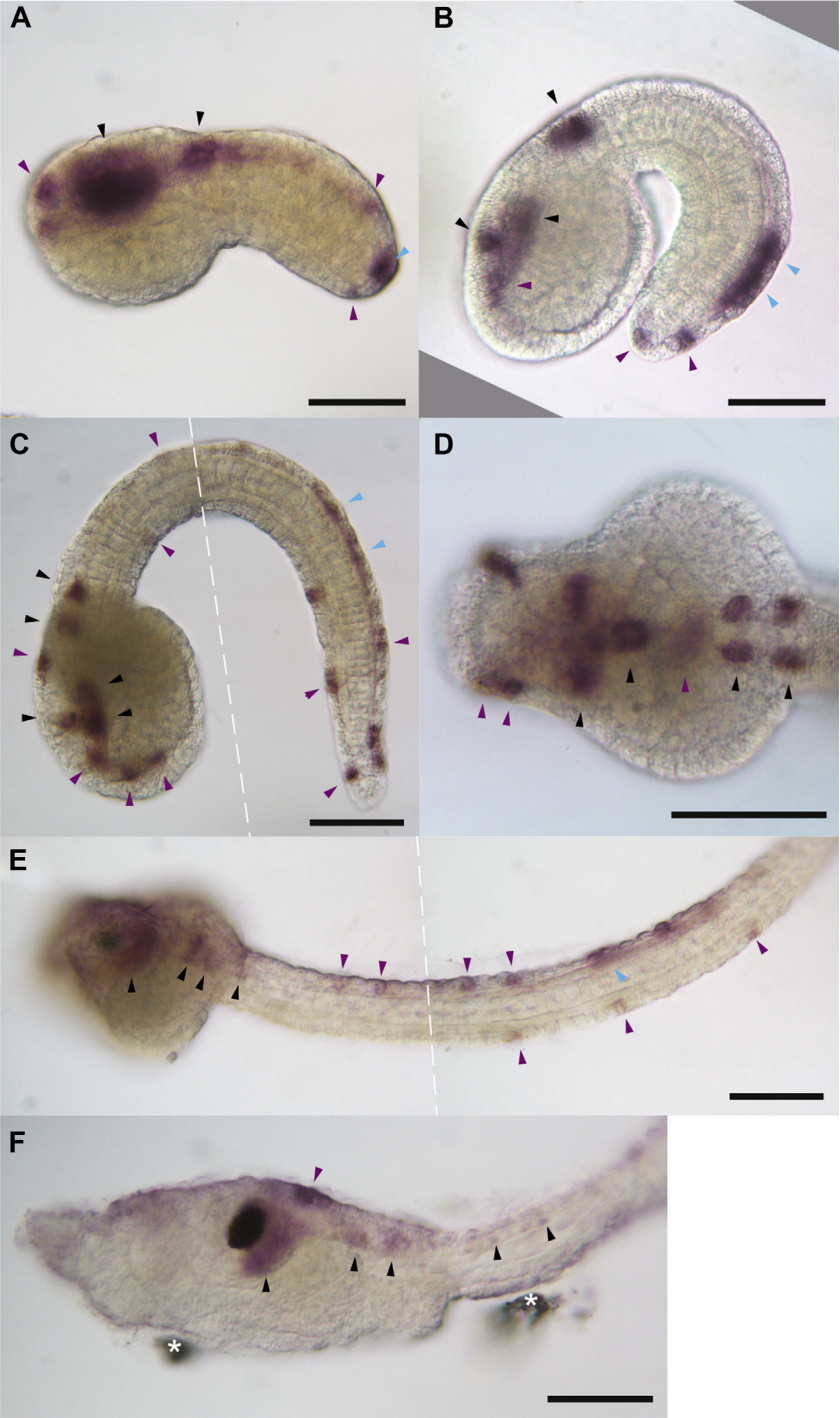
**Neuronal expression of CiNav1a in tailbud embryos and larvae of *Ciona*.** CiNav1a gene expression was detected by whole-mount *in situ* hybridization in *Ciona* tailbud embryos and larvae. These panels presented here were the images of samples from three independent trials. *Brown-colored stains* indicating the expression signals of CiNav1a are detected in the central and peripheral nervous systems. *A*, initial tailbud stage (*left-side view*). *B*, early tailbud stage (*left-side view*). *C* and *D*, mid tailbud stage (*left-side view* and *dorsal view*, respectively). *E*, late tailbud stage (*left-side view*). *F*, hatched larva (*left-side view*). All the specimens of embryos and larvae shown in the panels are oriented with anterior to the *left*. *Black arrowheads* indicate expression signal in presumptive neurons in the central nervous system of *Ciona* larvae. *Purple* and *cyan arrowheads* indicate epidermal sensory neurons and bipolar tail neurons, respectively, that constitute the peripheral nervous system of larvae. It is of note that all the stained neurons are not necessarily identified and labeled in the panels. The scale bars depict 50 μm. *Dashed lines* in *C* and *E* indicate the borders of combined two photos with different focal planes. *Asterisks* in *F* mark dusty materials unintentionally attached to the specimen. CiNav1a, ascidian *Ciona* Nav1.

**Figure 7:**
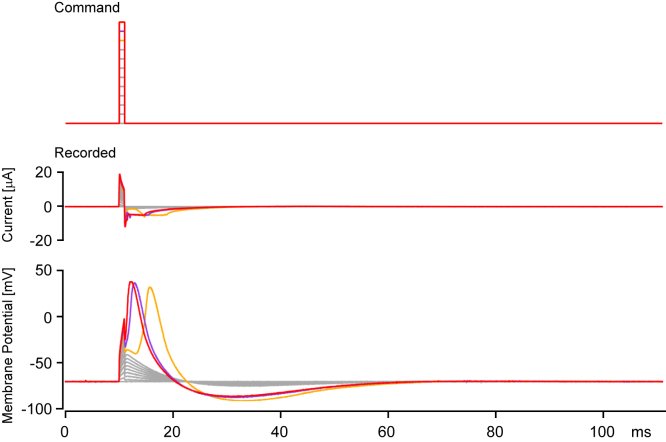
**Functional reconstitution of action potentials (APs) in *Xenopus* oocyte by coexpression of CiNav1a and CiKv1b.** Under the configuration of “loose” clamping at −70 mV (see Experimental procedures section), oocytes expressing CiNav1a and CiKv1b (without any auxiliary subunits) were stimulated with depolarizing step pulses (for 1 ms, 10 mV increment) (*top trace*). *Bottom traces* depict representative responses on membrane potential. APs were evoked by the stimulation; when larger pulse was applied, the latency of AP became shorter. *Orange*-, *purple*-, and *red*-colored traces indicate responses to 90, 100, and 110 mV depolarization pulse (for 1 ms), respectively. Similar APs were recorded from 38 cells. CiKv1b, one of shaker-related K^+^ channels in *Ciona*; CiNav1a, ascidian *Ciona* Nav1.

We examined the gene expression pattern of CiNav1a using whole-mount *in situ* hybridization. Corresponding to the expression pattern of *H. roretzi* TuNa1, which was exclusively expressed in larval neurons (12), CiNav1a expression signals were found in the cells of larval central and peripheral nervous systems, which include presumptive central neurons in the brain vesicle and motor ganglion, as well as peripheral neurons, such as epidermal sensory neurons in the surface of trunk and tail and bipolar tail neurons located in the peripheral nervous system (Fig. 6). In addition, CiNav1a mRNA was expressed in juvenile cerebral ganglion as examined by RT-PCR (data not shown). This is supported by mRNA expression profile in the expressed sequence tag database of *Ciona* (http://ghost.zool.kyoto-u.ac.jp/cgi-bin/fordetailht1.cgi?name=KY.Chr9.756.v1.SL1-1). Coexpression of CiNav1a with CiKv1b (one of shaker-related K^+^ channels in *Ciona*) in *Xenopus* oocyte showed action potentials (Fig. 7). These suggest that CiNav1a plays a substantial role in generating action potentials in ascidian neurons.

### The II–III linker of CiNav1a contains ABM, which can be targeted to the AIS

**Figure 8:**
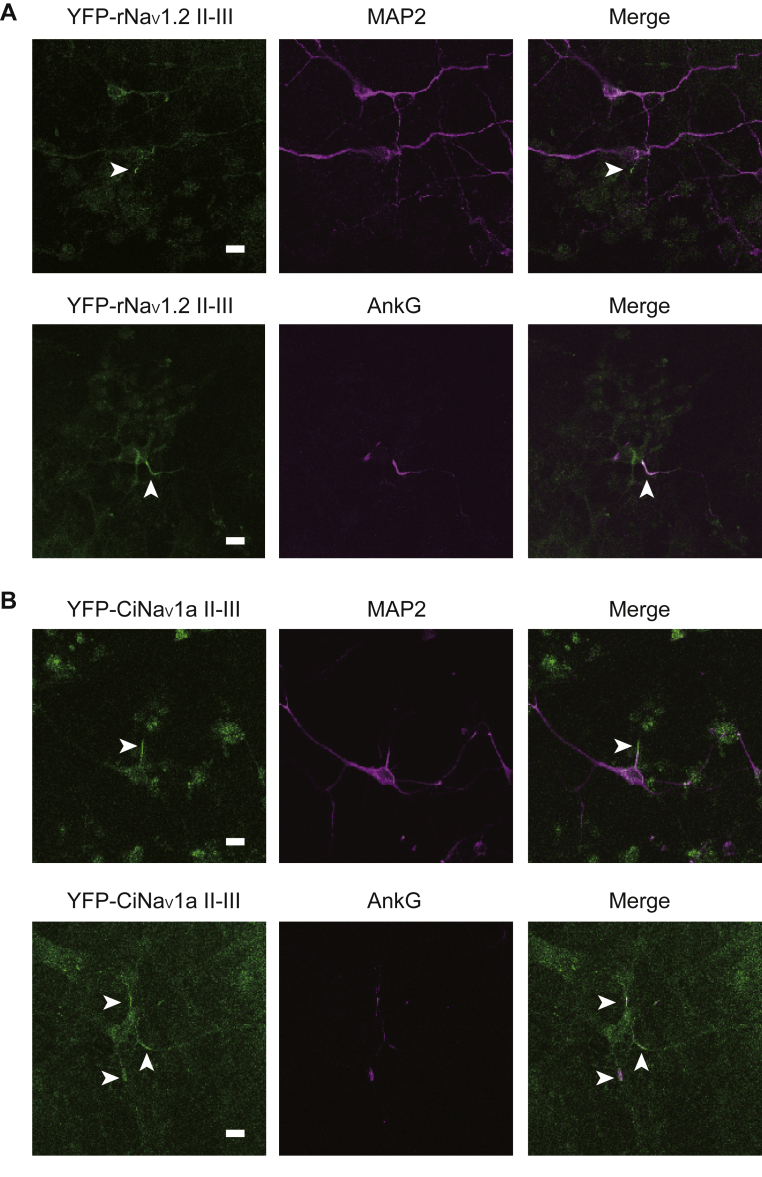
**Ability of targeting of the II–III linker of CiNav1a to the AIS of rat cortical neurons.***A*, *top*, rat cortical neurons expressing II–III linker of rNav1.2 fused with YFP was stained with the antibody to MAP2, a marker of dendrites and cell bodies. The II–III linker of rNav1.2 was localized in the proximity of MAP2 signal. The scale bar represents 10 μm. *Bottom*, rat cortical neurons expressing II–III linker of rNav1.2 fused with YFP was stained with the antibody to AnkG, a marker of AIS. The II–III linker of rNav1.2 was clearly colocalized with AnkG signal. The scale bar represents 10 μm. *B*, *top*, rat cortical neurons expressing II–III linker of CiNav1a fused with YFP was stained with the antibody to MAP2, a marker of dendrites and cell bodies. CiNav1a was localized in the proximity of MAP2 signal. The scale bar represents 10 μm. *Bottom*, rat cortical neurons expressing II–III linker of CiNav1a fused with YFP was stained with the antibody to AnkG, a marker of AIS. CiNav1a was clearly colocalized with AnkG signal. The scale bar represents 10 μm. Each data is a representative image in a single trial experiment. AIS, axon initial segment; CiNav1a, ascidian *Ciona* Nav1; MAP2, microtubule-associated protein 2; rNav1.2, rat Nav1.2.

Phylogenetic analysis of amino acid sequences from chordate Nav1s suggest that CiNav1a has an ABM in the II–III linker (27, 33) (Fig. 1*B*). To investigate the role of the ABM in CiNav1a, we expressed the II–III linker fused with YFP in the primary cultured rat cortical neurons by lentivirus. As a positive control, rat brain Nav1.2 II–III linker with YFP (Addgene; no. 91426) (37) was transfected. AIS was identified by positive signal of immunostaining with anti–ankyrin-G antibody (38, 39) and negative signal of immunostaining of anti–microtubule-associated protein 2 (anti-MAP2) antibody, which marks cell body and dendrites. CiNav1a–II–III linker–YFP signal overlapped with ankyrin-G signal (Fig. 8), which suggest that the II–III linker of CiNav1a can be targeted to AIS of rat cortical neurons.

## Discussion

In this study, we provide the first functional characterization of marine invertebrate Nav1, the ascidian Nav1 channel (CiNav1a) in a heterologous expression system. Although many marine invertebrate Nav1 cDNAs have been reported (10, 11, 12, 13, 14), any report of functional expression in heterologous cell system has not been available. One reason behind success of functional expression of CiNav1a could be that *Ciona* is closely related to vertebrates. However, our previous study of heterologous expression of *Halocynthia* Nav1a ortholog, TuNa1 (12), was not successful. At this moment, we do not know general rationale for success or failure of functional expression among invertebrate Nav1 channels.

CiNav1a showed rapid activation and inactivation without requiring the coexpression of auxiliary subunits. Forced coexpression of hβ1 did not affect channel properties. CiNav1a has a conserved ABM in the II–III linker, which drives the targeting to the AIS of mammalian neurons. Given that gene diversifications both of Nav1s and ankyrins occur after the branching point of ascidians and vertebrates (27, 40, 41), CiNav1a may provide evolutionary insight into vertebrate-type excitozones, such as the nodes of Ranvier and AIS (33).

### Unusual molecular properties of ascidian Nav1 channel; insensitivity to β subunits and TTX

We did not see effects of hβ1 on the gating kinetics of CiNav1a. In addition, CiNav1a was not affected by TipE. These findings are surprising given that it has been reported that the bacterial Nav channel can also be modified by mammalian β subunit (42). To explore why mammalian Nav1 β subunits fail to influence CiNav1a functions, we predicted a structure of CiNav1a bound to β1 by homology modeling (SWISS-MODEL) (8, 43, 44) based on cryo-EM structure of human Nav1.2–β2 complex (Protein Data Bank [PDB] ID: 6J8E) and electric eel EeNav1.4–β1 complex (PDB ID: 5XSY) (9). The model of CiNav1a structure was aligned to the EeNav1.4 molecule by PyMol in the EeNav1.4–β1 complex (9) (Fig. S2). Three residues of β1 subunit, E27, D31, R50, within the extracellular domain of the β1 subunit were identified important in the polar interaction with residues of EeNav1.4 (R1026 for E27, R1028 for D31, and K323 and D1484 for R50). On the other hand, only salt bridge from R50 was conserved in the model of CiNav1a–β1 complex where the counterpart of CiNav1a, D1850, corresponds to D1484 of EeNav1.4. A weak polar interaction is formed between the oxygen of K99 of β1 and amine of Q1893 of CiNav1a.

Both structures of EeNav1.4–Eeβ1 complex (9) and hNav1.4–hβ1 complex (7) suggest helix–helix interaction between the β1 transmembrane helix and the S0, S1, and S2 of domain III of α subunit. Residues in these segments are highly conserved in CiNav1a except a few changes (Fig. S2). Our model suggests that the interaction at the extracellular region, but not the interaction of transmembrane helices, is weaker in CiNav1a than in vertebrate Nav1s, which is consistent with our electrophysiology findings.

However, we cannot exclude the possibility of CiNav1a interacting with vertebrate β1, without the subsequent modification of gating. It has been reported that Nav1.5 and Nav1.8 expressed in heterologous expression system shows rapid gating without the need for coexpression of β subunits. Recently, a seminal study using voltage clamp fluorometry analysis of voltage sensor motion showed that motion of the voltage sensor motion of Nav1.5 is influenced by β subunits, although only to a minor degree of change to the current kinetics (45). It is possible that our recording in this study, even with the cut-open oocyte technique, is not sensitive enough to detect the change in the channel gating of CiNav1a by β subunits. Of note, we have yet to test other mammalian β subunits on CiNav1a. Further experiments are necessary to understand the molecular basis underlying the insensitivity of CiNav1a to β subunits.

Previous electrophysiological studies from *H. roretzi* embryos (46) and ascidian neurons (47) suggest that ascidian Nav channels are TTX insensitive. In the amino acid sequence of pore turret regions between S5 and S6 in domain IV of all TTX-sensitive Nav1s, a conserved acidic residue (TTSAGW**D**GLL) is found changed to alanine in both TuNa1 and CiNav1a (TTSAGW**A**GLL) (Fig. 1*A*). Since it has been reported that, in rat brain Nav1.2, mutation of this aspartic acid into glutamine makes rNav1.2 TTX insensitive (5) and also since TTX-resistant Nav1.4 of some group of garter snake, *Thamnophis sirtalis*, has mutation on the same site (48), we infer that this amino acid difference accounts for the TTX insensitivity in CiNav1a. Many marine invertebrate sodium channels are insensitive or resistant to TTX. Ascidians are filter feeders and feed on planktonic algae and microbes. Many Nav1 targeting toxins including TTX are produced by algae and microbes in oceans (49). It is possible that TTX insensitivity in ascidians or perhaps in other related species reflects an adaption necessary to their lifestyle as marine filter feeders. Such adaptations to sodium channel targeting toxins may be due to convergent evolution (48, 50, 51).

### CiNav1a is closely related to the ancestor of vertebrate Nav1 channels

Primary structures found in the vertebrate Nav1s, including the signature sequence important in sodium ion selectivity of pore region (D/E/K/A), the inactivation latch sequence of III–IV linker and the ABM in II–III linker are found conserved in CiNav1a. Importantly, CiNav1a is exclusively found in neurons, expressed in presumptive larval neurons as shown by whole-mount *in situ* hybridization and in the cerebral ganglion of juveniles as shown by our analysis with RT-PCR (data not shown). II–III linker of CiNav1a fused with YFP was targeted to the AIS in rat cortical neurons upon forced expression, suggesting that the II–III linker harboring ABM of CiNav1a has the ability to bind the giant ankyrin of mammalian neurons. Therefore, CiNav1a is a dominant neuronal Nav1 in *Ciona*, expressed in the larval central and peripheral neurons, similar to the previously reported TuNa1 of *Halocynthia* (12).

Two rounds of whole genome duplications are proposed to have occurred in the vertebrate lineage after the divergence from invertebrate chordates, which include ascidians (52). Diversification of Nav1 genes in vertebrates started from an ancestral gene, which represents the last common ancestor of CiNav1a gene and vertebrate Nav1 genes. The absence of CiNav1a expression in larval muscle is consistent with the finding that sodium spikes are not observed in ascidian larval muscle (53). For instance, Nav1.4 is specific to skeletal muscle and electric organ in vertebrates, and this gene diversity emerges after two rounds of genome duplications. These indicate that neuronal Nav1 is ancestral, and diversification thereafter by means of genome duplications and accumulated mutations underlies diversity of vertebrate Nav1 genes specialized for other organs than nervous systems, including cardiac muscle (Nav1.5), skeletal muscle (Nav1.4), and neural crest–derived sensory neurons in dorsal root ganglia that transduce nociceptive signals (Nav1.8 and 1.9) (27, 54).

**Figure 9:**
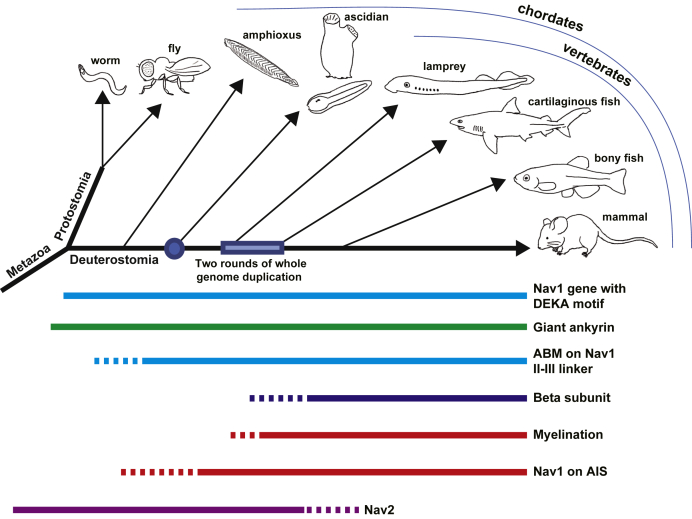
**Scheme of diversification and evolution of Nav1 and related events (β subunit genes, ankyrin binding).** Nav1 diverged before branch along chordate evolution. Ankyrin-binding ability predates appearance of β gene and myelin-related genes, and rapid conduction mechanisms were acquired serially through multiple steps. Nav1, voltage-gated sodium channel.

Two other Nav1 paralogs of *Ciona*, CiNav1b and CiNav1c, lack ABM. Inactivation latch sequence is only weakly conserved in both paralogs. Furthermore, CiNav1b has an altered sequence of the selectivity filter (D/E/T/E). Our molecular phylogenetic analysis suggests that gene duplications for such diversity seen in *Ciona* occurred after the appearance of the ancestral Nav1, which share common features with CiNav1a and vertebrate Nav1s, in which thereafter, the ABM was lost in the two orthologs, CiNav1b and CiNav1c. This is also the case with amphioxus, where multiple Nav1 paralogs exist, but only a single paralog contains ABM-like sequence in the II–III loop (27, 33) (Fig. 1*B*). In lamprey, two Nav1 paralogs that conserve ABM (XP_032802055 and XP_032831283 in Fig. 1*B*) are mainly expressed in the nervous system, and other three paralogs that only weakly conserve ABM show different expression patterns (27, 33, 55, 56). It was also noted that these lamprey Nav1 paralogs are not necessarily grouped with either of mammalian four Nav1 clades: Nav1.1/1.2/1.3/1.7, Nav1.4, Nav1.5/1.8/1.9, and Nav1.6 (27, 55, 56). These suggest that gene diversification from the CiNav1a-like ancestral Nav1 occurred independently in lamprey, and cell-type specific functional role was assigned to each gene, similarly to what presumably happened in the lineages of amphioxus, ascidians, and jawed vertebrates (55). Ideas on the evolutionary history of Nav1 with other biologically relevant events are summarized in Figure 9.

### Insights into evolutionary history of rapid conduction: Missing auxiliary subunit or extinction of the gene?

A previous study suggested that Nav β subunit can be traced to teleosts but neither in sharks nor lamprey (57). These suggest that innovation of modifying Nav1 gating and surface expression by β subunit occurred during the diversification of vertebrates much later than the branching point of vertebrates and ascidians (Fig. 9). We tested whether *Drosophila*'s TipE can influence the expression and properties of CiNav1a, but we did not observe any effect. It remains an open question whether ascidians contain any novel auxiliary subunit unique to ascidians that modulates the gating of CiNav1a, independent of either vertebrate β subunit or insect TipE. It is also interesting to see whether CiNav1a interacts with fibroblast growth factor 14-like molecule that is known to regulate gating and subcellular localization of Nav1s (58) in mammalian neurons.

The II–III linker of CiNav1a has a conserved ABM and that can be targeted to the AIS upon gene expression in rat cortical neurons. Giant ankyrin containing a region corresponding to the large exon is a critical innovation in vertebrate evolution of rapid conduction (39). *Ciona* genome contains a single ankyrin gene, expressed in larval neurons, that has a large exon and a highly similar exon–intron pattern to vertebrate ankyrins (40). A previous study described how an AIS-like structure is conserved in neurons of lamprey, which do not have myelin (33). *Drosophila* neurons also have AIS-like structures, but it does not cluster Nav1 channels (40). It is therefore interesting to explore whether Nav1 forms clusters by binding to ankyrin in ascidian neurons. In mammals, it has been shown that interspersed clusters of Nav1s along the axons before myelination or after demyelination facilitate rapid conduction. In *Aplysia*, Nav clusters in nonmyelinated nerves have increased conduction velocity compared with Nav homogenously distributed along the axons (59). It is an intriguing question whether addition of ABM to the II–III linker of Nav1 occurred in ancestral chordate, predating AIS establishment during chordate evolution. Future analysis of subcellular distribution of CiNav1a with relationship to the giant ankyrin in ascidian neurons is necessary to address such an important issue.

## Experimental procedures

All animal experiments were performed in accordance with regulations by the Animal Care and the Use Committee of Osaka University.

### Ciona

*C. intestinalis* (called type A), which Brunetti *et al.* (60) recently defined as *C. robusta*, were obtained from Misaki Marine Biological Station, The University of Tokyo (courtesy of Drs Satoe Aratake, Akihiro Yoshikawa, and Manabu Yoshida), or Graduate School of Science, Kyoto University (courtesy of Drs Reiko Yoshida and Yutaka Satou), through National BioResource Project, AMED, Japan. These adults were reared in laboratory tanks containing natural seawater or artificial seawater (*e.g.*, Marine Art BR). We surgically dissected the oviduct and spermiduct of the hermaphroditic adults and collected oocytes and sperm separately. Oocytes were inseminated by nonself sperm and allowed to develop in petri dish under room temperature (18–25 °C).

### cDNA cloning of a full-length *Ciona* sodium channel α subunit, CiNav1a

A cDNA pool was prepared by RT from total RNA isolated from hatched tadpole larvae of *Ciona* using PrimeScript RTase (TAKARA Bio, Inc) with oligo dT. Based on the genome information of *C. intestinalis* type A/*C. robusta* (XP_026691927), cDNA fragment covering the polypeptide fragment 1 to 1553 (amino acid number) of CiNav1a was amplified using forward and reverse PCR primers (primer 1 and primer 2 listed in Table S1) specific to CiNav1a with restriction site KpnI and XbaI, respectively, from the aforementioned cDNA pool. PCR products about 4.7 kb were extracted from agarose gel and subcloned into KpnI and XbaI sites of a modified version of pEF6–Myc-His (Thermo Fisher Scientific). A more 3’-sided rest fragment (2.3 kb) coding 1554 to 2325th of the deduced primary structure of CiNav1a followed by the subsequent 3’ UTR was amplified from the same cDNA pool using primer 3 and primer 4 (Table S1) and subcloned into the aforementioned plasmid at EcoRV and XbaI sites (pEF6–CiNav1a, 12.8 kb). For functional expression of CiNav1a protein in *Xenopus* oocytes, the full-coding region was amplified by PCR, keeping the KpnI site in the 5’-end of the translation initiator site and inserting a XhoI site (primer 5; Table S1) and subcloned into pCR4–TOPO by TA cloning using TOPO TA cloning kit (Thermo Fisher Scientific). A KpnI–XhoI fragment was excised out from this plasmid (pCR4–TOPO-CiNav1a, 11.0 kb) and then ligated into KpnI and XhoI sites of pSD64TR (gifted from Dr Terry Snutch, The University of British Columbia) (full-coding cDNA sequence of CiNav1a was deposited on DNA Data Bank of Japan (DDBJ)/GenBank under the accession no.: LC602262). For synthesis of complementary RNA (cRNA), this plasmid, pSD64TR–CiNav1a, was linearized by XbaI and incubated with SP6 RNA polymerase using mMESSAGE mMACHINE SP6 kit (Thermo Fisher Scientific).

The predicted full-length amino acid sequence of CiNav1a was compared with those of other Nav channels using MEGA7 and MEGA X (61, 62). Using 1148 amino acid positions without gaps, we constructed maximum likelihood molecular phylogenetic trees under Whelan and Goldman + frequency model. The tree with the highest log likelihood (−24718.31) was selected.

### Other cDNAs

Effects of auxiliary subunits on gating properties of CiNav1a were compared with those of rNav1.4, hNav1.5, and *Drosophila* Para. Plasmid for *Drosophila* Para was kindly provided by Dr Ke Dong (Michigan State University). Plasmids for hNav1.5, hβ1, and *Drosophila* TipE were kindly provided by Dr Mohamed Chahine (Laval University). rNav1.4 (also called μ1) was kindly provided by Dr Gail Mandel (Vollum Institute). Information for *in vitro* transcription with these plasmids (type of RNA polymerase and linearization enzyme) are as follows. hβ1 (pcDNA3) was digested by NotI and *in vitro* transcribed using T7 RNA polymerase. *Drosophila* Para (pGH19) was digested by NotI and *in vitro* transcribed using T7 RNA polymerase. *Drosophila* TipE (pSD64TF) was digested by XbaI and *in vitro* transcribed using SP6 RNA polymerase. μ1 was linearized by NotI and transcribed with T7 RNA polymerase. hNav1.5 (pcDNA3) was linearized by XbaI and transcribed with T7 RNA polymerase.

We obtained a full-coding cDNA sequence of a *Ciona* shaker-type K^+^ channel, CiKv1b (which corresponds to the gene model KY.Chr1.2378 at Ghost database [http://ghost.zool.kyoto-u.ac.jp/default_ht.html] and what was previously called Kv1.2 (24)) by 5'- and 3'-rapid amplification of cDNA ends using GeneRacer kit (Thermo Fisher Scientific), total RNA purified from tailbud stage embryos of *Ciona*, and gene-specific primers (primer 8–11; Table S1). Its coding sequence with the stop codon was amplified using primer 12 (CiKv1b–code-F–Kozak–XhoI) and primer 13 (CiKv1b–code-R–NotI) (Table S1). The amplified fragment was digested with XhoI and NotI and cloned into XhoI–NotI site of pSD64TF (also gifted from Dr Terry Snutch). The insert sequence encoding CiKv1b is deposited on DDBJ/GenBank under the accession no. LC600710. The plasmid was linearized with XbaI, and cRNA was synthesized using SP6 RNA polymerase.

### *Xenopus* oocyte expression

All electrophysiological studies were done in *Xenopus* oocyte. We tried expression of CiNav1a in HEK293T cells but failed to observe functional currents. *Xenopus* oocytes were harvested from animals anesthetized in water containing 0.2% ethyl 3-aminobenzoate methanesulfonate salt (Sigma–Aldrich). The oocytes were defolliculated by treating with type I collagenase (1.0 mg/ml; Sigma–Aldrich) in ND96 solution containing 96 mM NaCl, 2 mM KCl, 1.8 mM CaCl_2_, 1 mM MgCl_2_, 5 mM 4-(2-hydroxyethyl)-1-piperazineethanesulfonic acid (Hepes), 0.1 mg/ml gentamycin, 5 mM sodium pyruvate, pH  = 7.5 adjusted by NaOH. The defolliculated oocytes were then injected with cRNA synthesized using mMESSAGE mMACHINE Transcription Kit (Thermo Fisher Scientific).

### TEVC

Current recordings were conducted at 2 to 3 days after cRNA injection by using an amplifier (OC-725; Warner Instruments). Data were digitized by an AD/DA converter (InstruTECH LIH 8+8; HEKA Elektronik, at 10 kHz or Digidata1550A; Molecular Devices at 20 kHz), acquired with PatchMaster software (HEKA Elektronik) or pClamp10.5 (Molecular Devices). The data were analyzed with pClamp10.5 or IgorPro (WaveMetrics). ND96 was used as bath solution. Glass electrodes had resistance of 0.3 to 0.5 M Ω after filling with 3 M KCl solution. TTX (FUJIFILM Wako) was dissolved in H_2_O and frozen as 1 mM stock solution.

### Cut-open oocyte voltage clamp

Cut-open oocyte voltage clamp recordings were conducted as previously described (25). Briefly, current recording was conducted at 1 to 2 days after cRNA injection by using an amplifier (CA-1B; Dagan). Current output signals were low-pass filtered at 10 kHz through the built-in four-pole Bessel filter, then digitized at 50 kHz by an AD converter (Digidata1440A; Molecular Devices), and analyzed with pClamp10.1 (Molecular Devices). Extracellular solution contained 105 mM NaMeSO_3_, 20 mM Hepes, and 2 mM Ca(OH)_2_. pH was adjusted to 7.4 with methanesulfonic acid. Intracellular solution contained 105 mM *N*-methyl-d-glucamine, 20 mM Hepes, and 2 mM EGTA. pH was adjusted to 7.4 with methanesulfonic acid. The oocyte was permeabilized with 0.1% saponin (Sigma–Aldrich).

### “Loose” oocyte clamping

To examine whether CiNav1a can generate action potentials, the method called “loose” oocyte clamping (63) was applied. Mixed cRNAs for CiNav1a (55 ng/μl) and CiKv1b (10 ng/μl) were injected into defolliculated *Xenopus* oocytes. To obtain action potential recordings, we inserted a simple circuit of a 50 MΩ resistor as well as a series of a diode and a 10 MΩ resistor between the oocyte and the current electrode of OC-725C (Warner Instruments) (63). This circuit allows the TEVC amplifier to effectively depolarize the oocyte membrane potential through the smaller resistor but not to easily repolarize it because of the inserted larger resistance (“loose” clamping (63)). Experiments were performed at about 20 to 22 °C. Current and voltage glass electrodes (filled with 3 M KCl, 0.05–0.4 MΩ) were inserted into an oocyte soaked in ND96. Under the “loose” clamp configuration at −70 mV, 1 ms voltage pulses were applied. The membrane potential and current changes were recorded by an AD/DA converter (ITC-16) and software (Pulse; HEKA Electronik) that ran on a Windows PC.

### Analysis of electrophysiology data

The recorded data were analyzed using IgorPro. Steady-state inactivation was fitted by the equation: 1/{1+exp(V − V_half)/*k*} where V_half is the voltage that gives half value and *k* is the slope factor. Current decay of rNav1.4 and CiNav1a and recovery from inactivation of CiNav1a were fitted by single exponential function. Recovery from inactivation of hNav1.5 was fitted by double exponential function. The data were statistically analyzed using *t* test or two-way repeated-measures ANOVA as appropriate. Values are shown as mean ± SD.

### Whole mount *in situ* hybridization of *Ciona* embryo

Fertilized eggs of *Ciona* were dechorionated using seawater whose pH was elevated to 12 by NaOH and also containing a protease (0.5 mg/ml actinase E; Kaken Pharmaceutical Co Ltd) and thioglycolic acid (10 μl/ml) (WAKO). After several washes with fresh artificial seawater, dechorionated embryos were placed on agar-coated dish and allowed to develop in the artificial seawater. Tailbud embryos and larvae were fixed with 4% paraformaldehyde in 0.5 M NaCl and 0.1 M Mops (pH 7.5) overnight at 4 °C and were stored in 80% ethanol below −20 °C before use.

The full-length cDNA clone of CiNav1a was linearized, and the antisense probes were prepared using digoxigenin (DIG)-labeling mix (Roche) and T3 RNA polymerase (Roche) by standard methods. The synthesized RNA probe was fragmented to ∼300 bp by alkaline treatment. Whole-mount *in situ* hybridization was performed according to the protocol described previously (64) with minor modifications. The fixed specimens were rehydrated by thorough washes with PBST (PBS containing 0.1% Tween-20) and then partially digested with proteinase K in PBST (1.5 μg/ml for early and mid-tailbud embryos, 2 μg/ml for late-tailbud embryos, and 3 μg/ml for hatched larvae) for 40 min at 37 °C. After several washes with PBST, specimens were postfixed with 4% paraformaldehyde in PBST for 1 h at room temperature (20–25 °C). After several washes with PBST, they were prehybridized with hybridization buffer containing 50% formamide, 5× saline sodium citrate (SSC) buffer, 5× Denhardt's solution, 0.1 mg/ml (for early and mid-tailbud embryos), or 1.5 mg/ml (for late-tailbud embryos and hatched larvae) yeast tRNA, 2% dextran sulfate, and 0.1% Tween-20 for 2 h at 50 °C. Thereafter, they were soaked in the hybridization buffer containing the DIG-labeled probe (0.1 μg/ml for early and mid-tailbud embryos, 0.01 μg/ml for late tailbud embryos and hatched larvae) for 2 days at 50 °C. The hybridized embryos/larvae were washed twice with 2× SSC, 50% formamide, 0.1% Tween-20 at 50 °C for 15 min, and then the excessive probes were digested with RNase A (20 μg/ml) in RNase reaction buffer containing 0.5 M NaCl, 10 mM Tris–Cl (pH 8.0), 5 mM EDTA, and 0.1% Tween-20 for 30 min at 37 °C. Then specimens were washed in RNase reaction buffer once, washed once in 50% formamide, 2× SSC, and 0.1% Tween-20 at 50 °C for 20 min, and then twice in 0.5× SSC, and 0.1% Tween-20 at 50 °C for 15 min. After thorough replacement with PBST, the specimens were soaked in a blocking solution (0.5% [w/v] blocking reagent [Roche] in PBST) for 30 min at room temperature and then in the 1/2000 alkaline phosphatase (AP)–conjugated anti-DIG antibody (Fab fragment; Roche) in the blocking solution for overnight at 4 °C. The specimens were rinsed thoroughly by PBST, and then the signal was detected with standard nitro blue tetrazolium/5-bromo-4-chloro-3-indolyl phosphate staining for AP. After washes with PBST, the stained specimens were observed under a differential interference contrast microscope (LWD CDPlan 40×, numerical aperture 0.55, mounted on IMT-2, Olympus) and pictured using a digital camera (DS-Fi2; Nikon).

### Production and titration of lentiviral vector carrying a gene encoding the YFP-fused II–III linker of CiNav1a

The domain II–III linker, consisting of 220 amino acids, from 984S to 1203K of rat brain type 2 sodium channel (rNav1.2) in the plasmid of pl-Synapsin-YFP-rNaV_II–III (Addgene; no. 91426 (37)) was replaced by the corresponding region, from 1112S to 1351R, consisting of 240 amino acids of CiNav1a (designated as pl-Synapsin-YFP-CiNav1a_II–III).

Lentiviral particles containing pl-Synapsin-YFP-rNaV_II–III (positive control plasmid carrying a cDNA fragment corresponding to the domain II–III linker of rNav1.2 channel fused with YFP downstream the synapsin promoter) (https://www.addgene.org/91246/; Addgene; no. 91426) or pl-Synapsin-YFP-CiNav1a_II–III (test plasmid) were generated by cotransfecting either of these with pl-Synapsin-YFP-CiNav1a_II–III or pl-Synapsin-YFP-rNaV_II–III with pLP1 plasmid (containing the gag/pol), pLP2 plasmid (encoding the Rev protein), and pLP/VSVG (encoding the envelope protein) into HEK293T in BSL-2 level laboratory of Graduate School of Medicine, Osaka University. Culture medium was collected at 48 h after transfection once. The supernatant was centrifuged at 19,000 rpm for 140 min using Avanti-HP301, A-30,50Ti. The viral vector pellet was resuspended in Dulbecco's modified Eagle's medium, aliquoted, titered, and kept frozen at −80 °C.

### Culture of rat brain cortical neurons, expression of the II–III linker of CiNav1a fused with YFP and immunohistochemistry

Primary cortical neurons were prepared from Wistar rats on embryonic day 18 as previously described (65). Pregnant rats were deeply anesthetized with isoflurane and decapitated. The uteri were removed and placed in ice-cold PBS. The dissociated neurons were obtained by digesting cortex with the solution containing 0.35% (w/v) Papain (Wako; 0.5 unit/g), 0.05% (w/v) EDTA (Dojin) in PBS(−) at pH 7.4 for 30 min at 37 °C followed by the brief treatment with DNaseI in modified Eagle's medium and mechanical trituration through Pasteur pipettes. Neurons were plated at 0.5 × 10^6^ on poly-l-lysine-coated cover glass (diameter = 18 mm; Matsunami Glass Industries Ltd). Transfection was performed at 7 days. The cortical neurons cultured *in vitro* were infected with 2.5 × 10^4^ to 1 × 10^5^ pfu/ml of lentiviral vectors with multiplicity of infection = 1 carrying either YFP-CiNav1a_II–III or YFP-rNaV_II–III in serum-containing Dulbecco's modified Eagle's medium for 24 h. Medium was changed 6 h later. Cultures were continued for 3 days and then fixed for microscopy observation. Neurons were fixed by 4% formaldehyde/PBS at 4 °C for 10 min. After the permeabilization with PBST (0.2%), they were incubated with blocking buffer consisting of 5% goat serum in PBS for 1 h at room temperature. Immunostaining of ankyrin-G and MAP2 was performed using anti-ankyrinG antibody with 1:500 dilution (N106/65; UC Davis/NIH NeuroMab Facility) and anti-MAP2 antibody with 1:500 dilution in PBS (AP-20; ab11268; Abcam) with 1 h incubation followed by the secondary antibody Alexa Fluor 647 antimouse antibody (Invitrogen) with 1:1000 dilution in PBS for 1 h.

Stained neurons were observed under fluorescence microscope, FV1200 (Olympus) with UPlanSApo 100×/1.35 Sil∞/0.13-0.19/FN22.

### Homology modeling of protein structure of CiNav1a

The protein structure of CiNav1a was predicted by homology modeling using SWISS-MODEL server (43, 44, 66) based on cryo-EM structure of human Nav1.2–β2 complex (PDB ID: 6J8E) (8). Nav1.2 was used as a template since Nav1.2, rather than Nav1.4, is closer to CiNav1a (53% amino acid sequence identity). The model was aligned to the EeNav1.4 molecule–β1 in the EeNav1.4–β1 complex (PDB ID: 5XSY) (9) by PyMol.

## Data availability

cDNA sequences of CiNav1a and CiKv1b were registered in DDBJ/GenBank under the accession no. LC602262 and LC600710, respectively. All other data relevant to this study are included within this article.

## Supporting information

This article contains supporting information.

## Conflict of interest

The authors declare that they have no conflicts of interest with the contents of this article.
