# Supplementary material for: Heterologous functional expression of ascidian Nav1 channels and close relationship with the evolutionary ancestor of vertebrate Nav channels
Source: J Biol Chem. 2021 May 14;296:100783. doi: 10.1016/j.jbc.2021.100783 (PMC8192821; doi:10.1016/j.jbc.2021.100783)
Supplement: Supplemental Figures S1 and S2 [file mmc1.docx]

Supporting information

Heterologous functional expression of an ascidian Nav1 channel with close relationship with the evolutionary ancestor of vertebrate Nav channels

Authors: Takafumi Kawai^1^, Masaki Hashimoto^2^, Natsuki Eguchi^3^, Junko M. Nishino^4,5^, Yuka Jinno^1#^, Risa Mori-Kreiner^1^, Måns Aspåker^6^, Daijiro Chiba^4^, Yukio Ohtsuka^7^, Akira Kawanabe^1^**, Atsuo S. Nishino^4,5^, Yasushi Okamura*^1,2^

From the ^1^Integrative Physiology, Department of Physiology, Graduate School of Medicine, Osaka University, ^2^Graduate School of Frontier Bioscience, Osaka University, ^3^UC Davis, ^4^Department of Biology, Faculty of Agriculture and Life Science, Hirosaki University, ^5^Department of Bioresources Science, United Graduate School of Agricultural Sciences, Iwate University, ^6^Uppsala university, ^7^Biomedical Research Institute, National Institute of Advanced Industrial Science and Technology (AIST), 1-1-1 Higashi, Tsukuba, Ibaraki 305-8566, Japan

*To whom correspondence should be addressed; Yasushi Okamura, MD, PhD., Integrative Physiology, Department of Physiology, Graduate School of Medicine, Osaka University, Yamada oka 2-2, Suita, Osaka, 565-0871, Japan, TEL +81-6-6879-3310, FAX +81-6-6879-3319, [vsop1@me.com](mailto:vsop1@me.com)

# Present address; Department of Medical Biochemistry, Graduate School of Medicine, Osaka University, Yamada-oka, 2-2, Suita, Japan

** Present address; Molecular Physiology & Biophysics, Faculty of Medicine, Kagawa University, miki-cho, Kagawa, Japan

Content: Figures S1, S2

Figure S1


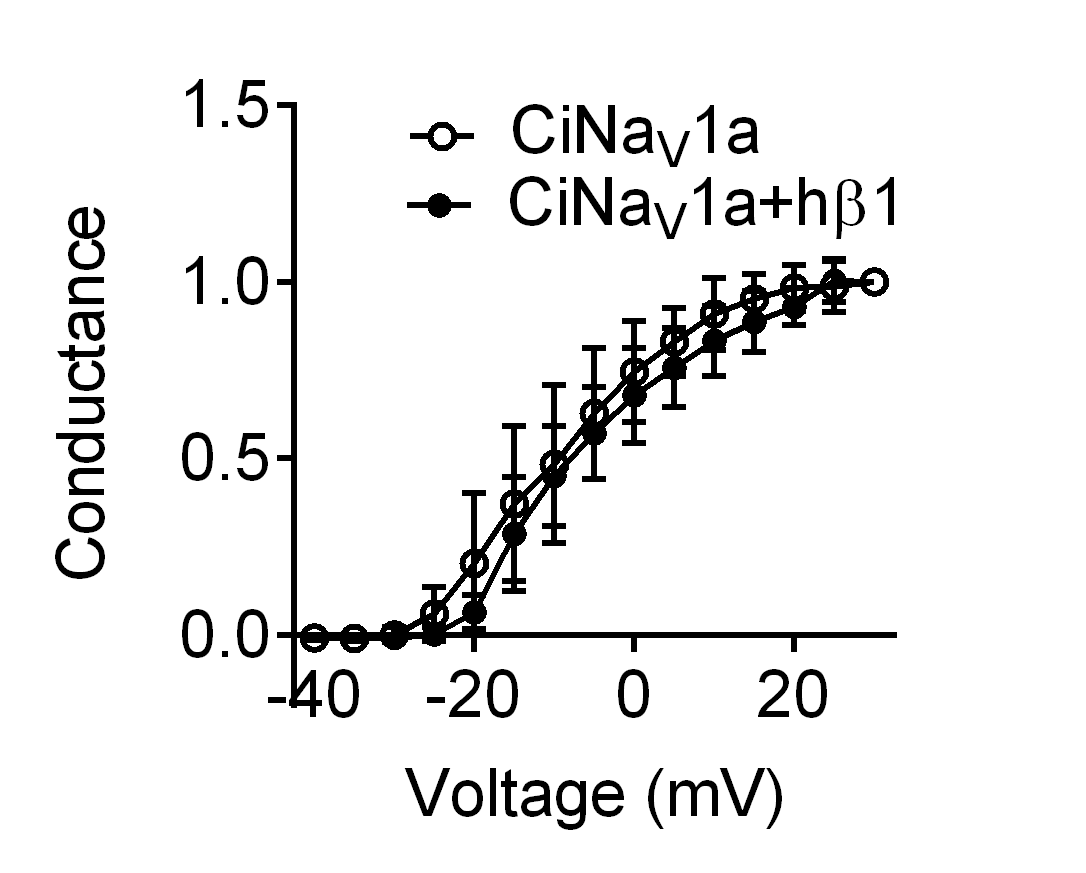
Supplementary Figure 1

G-V curves of CiNav1a with or without hβ1.

G-V relationship of CiNav1a was converted from the I-V curve obtained in Fig. 3B. The reversal potential was estimated by extraporation from the data of I-V curve. N=7, and 5 for CiNav1a alone and CiNav1a with hβ1, respectively. We did not observe significant difference (P= 0.2907 as analyzed by two-way ANOVA), which is consistent with the I-V curve data. The graphs present mean ± SD

Figure S2


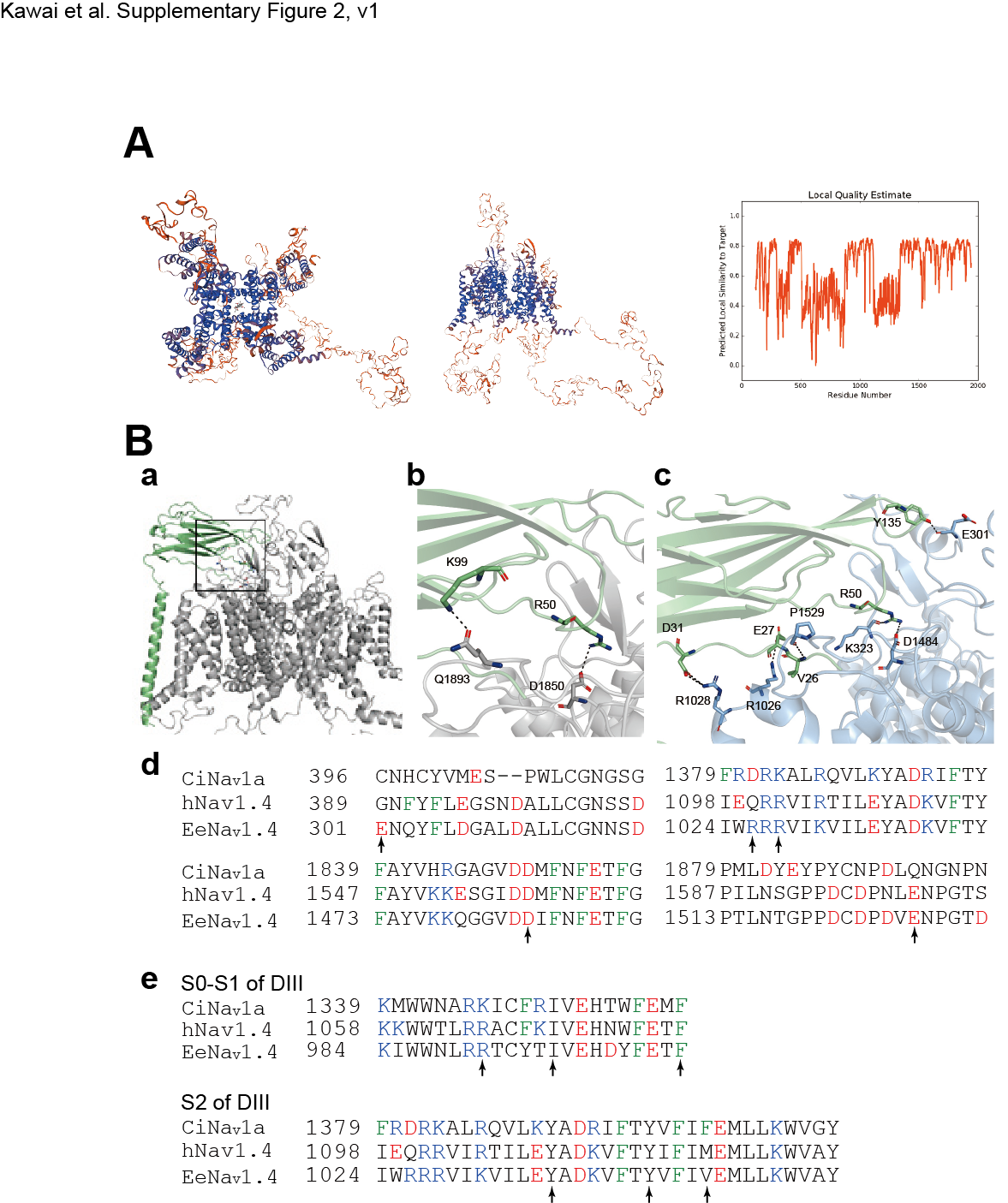


Supplementary Figure 2

Predicted protein structure of CiNav1a with electric eel β1(Eeβ1) by homology modeling and docking.

A, Left, Predicted structure of CiNav1a using human Nav1.2 as template by SWISS-MODEL server. CiNav1a model was built using hNav1.2 as a template since Nav1.2, rather than Nav1.4, is closer to CiNav1a (53% amino acid sequence identity). Blue denotes a local QMEAN >0.6 (high quality), orange denotes a local QMEAN <0.6 (low quality). Right, Local QMEAN for each residue of CiNav1a. B, (a) Side view of the CiNav1a (grey) + Eeβ1 (green) complex created in Pymol, top is extracellular side. Square corresponds to the area shown in (b) and (c). Interacting residues shown as sticks. (b) The two polar bonds found between CiNav1a and β1-subunit. (c) The multiple polar bonds found between EeNav1.4 (light blue) and Eeβ1. (d) Sequence alignment of extracellular interfaces of EeNav1.4, CiNav1a and hNav1.4. (e) Sequence alignment of EeNav1.4, CiNav1a and hNav1.4 at β1-subunit facing transmembrane regions consisting of S0, S1 and S2 of domain III. (d) and (e), arrow indicates site that was shown to interact with Eeβ1 by the cryo-EM structure.
